# Knowledge and attitudes toward nutritional deficiencies in celiac disease among medical students and healthcare providers in Egypt

**DOI:** 10.1038/s41598-025-12247-5

**Published:** 2025-07-31

**Authors:** Mohammed N. Abdelaziz, Hajer Azzam, Abdalla Hefnawy, Ahmed R. A. Moustafa, Elkasabi Team, Hager G. Elkasabi, Abdelmalek Elkasabi, Omar Abdallah

**Affiliations:** 1 Faculty of Medicine, Mansoura University, Mansoura, Egypt; 2 Manchester Program, Faculty of Medicine, Mansoura University, Mansoura, Egypt; 3 Lecturer of Hepatology and Gastroenterology, Faculty of Medicine, Mansoura University, Mansoura, Egypt

**Keywords:** Knowledge, Attitude, Celiac disease, Healthcare providers, Medical students, Nutritional deficiency, Egyptian, Gastroenterology, Health care, Medical research, Risk factors, Signs and symptoms

## Abstract

Celiac disease is an autoimmune enteropathy caused by gluten intake in foods and cereals. Weight loss, persistent diarrhea, and starvation are all possible outcomes. A lack of awareness about CD and malnutrition among medical students and healthcare professionals can result in inadequate nutritional guidance and delayed diagnoses, ultimately compromising patient outcomes and treatment adherence. Greater education and training programs for medical students can help alleviate these concerns and enhance patient outcomes. This study aims to assess the level of knowledge, attitude, and practice regarding nutritional deficiencies related to celiac disease among medical students and healthcare providers in Egypt. The secondary outcome is to identify knowledge gaps and potential barriers to effective implementation. This was a questionnaire-based cross-sectional study. The targeted population included students enrolled in any Egyptian medical institution during the academic year 2024–2025, as well as healthcare providers. The questionnaire included sections on demographic information, knowledge about celiac disease and nutritional deficiencies, and attitudes towards nutritional management of celiac disease. Using a convenient sampling method, it was distributed electronically in the English language. A total of 1233 people participated in the study. The median knowledge of celiac disease was 13, indicating that only 11% of individuals had adequate understanding of the condition (8–19). The median knowledge of women and those who lived in cities was higher (*p* = 0.003 and ←0.001, respectively). Physicians in internal medicine had a greater median level of expertise than pediatricians, indicating a significant difference in specialty among healthcare practitioners (*p* = 0.01). Only 11.9% of participants demonstrated sufficient understanding of the nutritional inadequacies linked to a gluten-free diet. The median knowledge of women and non-Egyptian individuals was higher (*p* = 0.05 and 0.03, respectively). Different governorate regions had significantly different knowledge medians (*p* = 0.002), with Delta governorates having a higher median than Greater Cairo (*p* = 0.004). This study highlighted the gap in CD knowledge among Egyptian medical students and healthcare providers, particularly concerning symptoms, diagnostic methods, and dietary management.

**Supplementary Information:**

The online version contains supplementary material available at 10.1038/s41598-025-12247-5.

## Introduction

A gluten-induced autoimmune enteropathy, celiac disease (CD), arises in genetically predisposed individuals^1^. It is induced by ingesting gluten, a group of glycoproteins in certain processed foods and cereals^2^. Thus, innate and adaptive immune systems are activated in a hyperimmune response evoked by gluten ingestion^3^. Systemic cytokine release contributes to some of the extraintestinal symptoms of celiac disease. These autoantibodies and inflammatory cytokines in the mucosa lead to mucosal damage^3,4^. Intestinal villous flattening from mucosal autoimmunity in celiac disease significantly decreases the absorptive surface area for nutrients and causes villous atrophy^5^. Certain of the clinical features of CD include weight loss, chronic diarrhea, and various nutritional deficiencies. Additionally, extraintestinal manifestations in the form of anemia, osteopenia, coagulopathy, and neurological symptoms have been reported^6,7^. Although nutrition plays a pivotal role in CD management, medical students and healthcare providers (HCPs) often have inadequate knowledge and awareness of the nutritional deficiencies associated with CD. In previous research conducted in several countries, it has been demonstrated that a significant proportion of healthcare professionals (HCPs) possess limited knowledge about celiac disease (CD) and the gluten-free diet^8,9^. Failure to identify nutritional deficiencies of CD was also observed in other research on health and medical students^10,11^. The lack of awareness could lead to inappropriate dietary advice and delayed diagnosis of CD patients, which can affect patient outcomes and treatment adherence^12,13^. CD is not an uncommon disorder among Egyptian children. In a study of 1,500 children from the general pediatric population, 8 children had CD^14^. The prevalence in this population was approximately 0.53% (1 in 187)^14^. Thus, CD is emerging as a public health problem in Egypt. Little is known regarding medical students’ knowledge and attitudes about the disease and the nutritional disorders with which it is linked. Education and training campaigns for healthcare providers would be able to tackle nutritional deficiencies related to CD^15^. To minimize the risk of late diagnosis and complications, Abu-Zekry et al.^14^ also recommend that Egypt should urgently establish a case-finding policy and increase awareness of CD. As future physicians, medical students can have a significant role in the early diagnosis, treatment, and education of celiac disease patients. Outcomes of patients can be significantly influenced by the extent of their knowledge of nutritional deficiencies of the condition. Accordingly, the research aims to assess HCPs’ knowledge gaps regarding nutritional deficiencies of the CD and improve the curriculum. This will enable the future HCPs to provide holistic care for CD patients. This will also inform targeted educational interventions and curricula to enhance medical students’ preparedness to optimally manage celiac disease and achieve better patient outcomes.

## Procedures and materials

### Population and study design

**Fig. 1:**
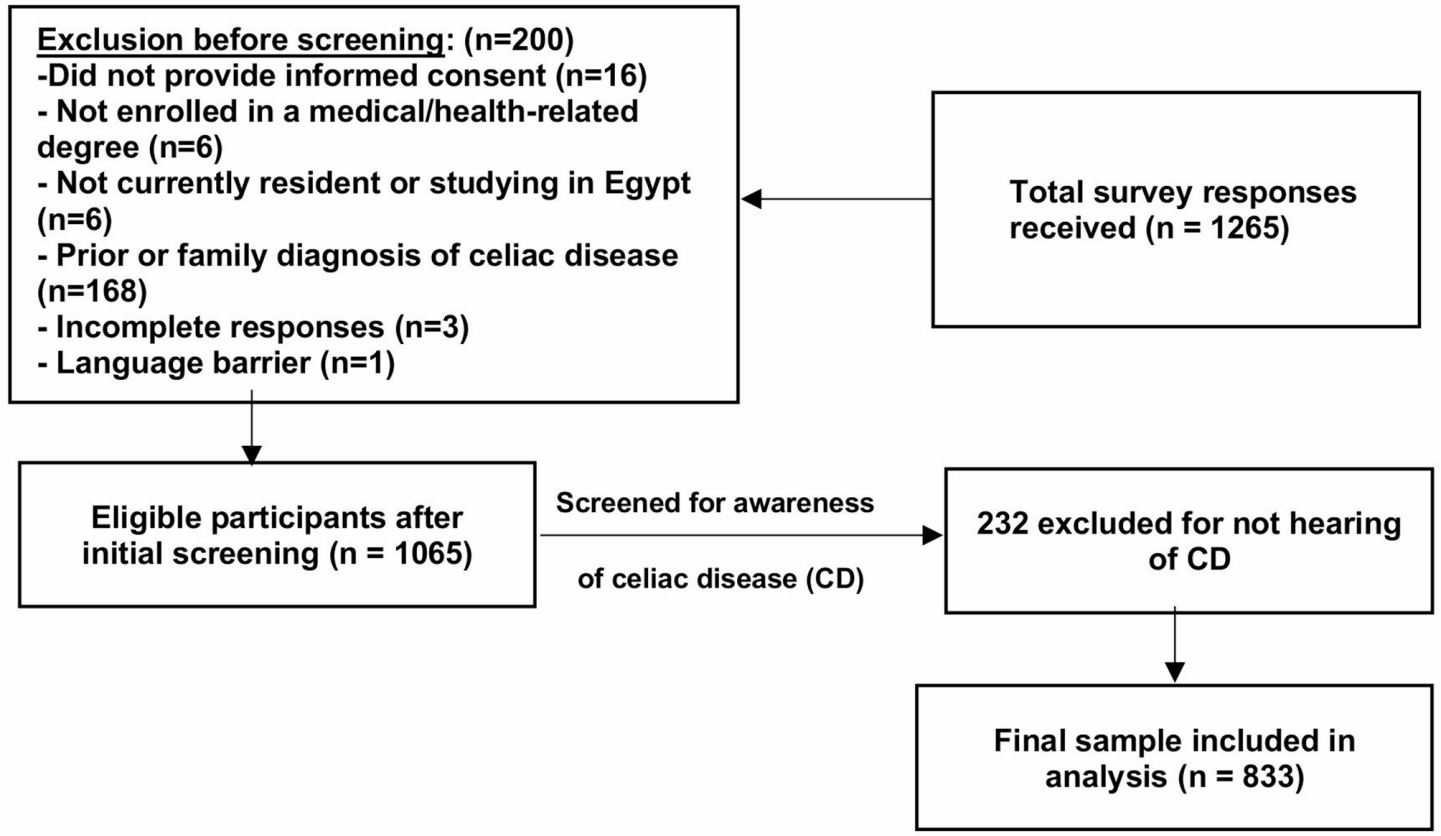
A flowchart illustrating participant flow and response exclusion.

We employed a questionnaire-based data collection strategy in Egypt. The questionnaire was distributed in English. Specifically, from November 2024 to February 2025, the group under study included students enrolled in any Egyptian medical institution during the academic year 2024–2025, as well as healthcare providers. For healthcare providers, they were licensed medical professionals who were involved in clinical care, particularly in fields related to gastroenterology, internal medicine, pediatrics, or general practice. Students enrolled in the first and second academic years were known as “preclinical students.” We categorized third-, fourth-, and fifth-year students as “clinical students.” Exclusions would be made of those participants not enrolled in a medical or health-related degree program, those failing to provide informed consent or withdrawing at any time, and medical students not currently resident or studying in Egypt. Students with a prior diagnosis of celiac disease or currently being treated for it are excluded to prevent bias in measuring knowledge and attitude. Family and personal history, as well as the ability to gain personal experience and manage diet daily, could be potential confounders, making it harder to ascertain information gleaned from education in medicine and direct experience. By excluding those with family or individual celiac disease, the study is better able to measure the baseline attitudes and knowledge of medical students and physicians without direct contact. Those who did not fully respond to the questionnaire or were unable to provide a decent amount of data were excluded from the final analysis. Lastly, students who faced language problems, preventing them from understanding and responding to the survey, were excluded (Fig. 1). The sample size was determined online (http://www.openepi.com/SampleSize/SSPropor.htm). Based on Dembiński, Lukasz et al.^10^ (p), a 5% margin of error, and a 95% confidence interval, a minimum sample size of 538 is necessary. By factoring in a 40% predicted drop rate, the required sample size is 753. A convenient sample strategy was used to recruit participants. Each group member sends the questionnaire using an instant messaging technology such as WhatsApp, Facebook, or Messenger Inc., along with a direct hyperlink generated by Google Inc.‘s survey management tool Forms. Before transmitting the online form to the web server, participants carefully completed all of the questions. To overcome potential biases, we implemented clear communication about the survey’s purpose and reminders to encourage participation. The Declaration of Helsinki was followed in all study procedures. The study was designed and reported under the STROBE (Strengthening the Reporting of Observational Studies in Epidemiology) guidelines. A complete STROBE checklist is provided as Supplementary Material 1.

### Questionnaire deployment

A panel of public health and gastroenterology experts conducted a review of current literature^10,11,13^ to determine the validity of the questionnaire. A three-section structured questionnaire included 34 items. It consists of demographic information, knowledge about celiac disease, and attitudes toward celiac disease nutritional deficiencies. We conducted pilot research with 45 participants from the target population to confirm the ease and clarity with which the questionnaire was completed. We subsequently excluded the findings of this pilot trial from the final analysis. The questionnaire was divided into three components that were designed to assess celiac disease knowledge and attitudes, as well as nutritional deficits. *The first sector* assessed the participants’ demographics and variables, including age, gender, marriage status, geographic location (rural or urban), nationality, economic status, governorate, and occupational and professional information. Following the first sector, we asked participants, “Have you or any of your family members been diagnosed with celiac disease?” to determine their baseline level of knowledge. Participants who answered “no” were sent to the second leading question, “Have you ever heard about celiac disease?” Participants who responded positively were sent to the second and third sections. *The second sector* assessed knowledge and attitudes about celiac disease. The knowledge questions consisted of five questions, each with one correct answer for one point. The other four questions included multiple answers for each question and awarded one point for each correct answer. The maximum score was 38 points. A score of 22.8 (60%) was established as the threshold for acceptable and unsatisfactory levels of knowledge^10,11,13^. Considering the variety of unavailable resources, the question on the methods that can be utilized to analyze CD permitted multiple choice without awarding points for responses. There was also a question about their desire to learn more about CD and the type of information they want. This question likewise offered many options without assigning points. *The third sector* evaluated the knowledge of gluten-free diets and their associated nutritional deficiencies. It included six yes or no questions and three questions allowing multiple responses, giving one point for each correct answer. The total maximum score was 21 points. A score of 12.6 (60%) was set as a cut-off value for acceptable levels of knowledge. There were also two 5-scale questions without additional scores asking about their thoughts on the wide availability of gluten-free diets and the ability to manage them without a dietitian’s help.

### Statistical analysis

IBM-SPSS software (IBM Corp., released 2020). IBM SPSS Statistics for Windows, Version 27.0. Armonk, NY: IBM Corp.) was used for statistical analysis. Categorical variables were described as frequencies and percentages. Continuous variables were described as the median and interquartile range (IQR), as they were not normally distributed using Quantile-Quantile (Q-Q) plots. Continuous data were compared using the Mann-Whitney U test and the Kruskal-Wallis H test. Spearman’s correlation coefficient was used for the correlation between continuous variables. Linear regression was used to determine predictors of knowledge. A p-value of 0.05 or less was considered statistically significant. For regression analyses, we calculated both the R² and adjusted R² values to evaluate the proportion of variance explained by the models while accounting for the number of predictors.

## Results

### Sociodemographic analysis

Table 1 shows the sociodemographics of participants. Our survey comprised 1233 participants, of whom 59.5% were men, 95.3% were younger than 30, and 88.3% were Egyptian. 72.6% were from Delta Governorates, and 17.8% were from Greater Cairo. Regarding their residence and marital status, 69.7% were from urban residences, and 92.4% were single. 79.8% and 13.9% expressed an average and high economic level, respectively. The majority were undergraduate students, 60.8% clinical students, and 12.8% preclinical students. Of 325 healthcare providers, 50.5% were family medicine physicians, 25.8% were internal medicine physicians, 19.1% were pediatricians, and 4.6% were interns. The majority reported their places of work as follows: 53.5% city hospitals, 17.5% district hospitals, and 7.4% private outpatient hospitals. 74.2% had work experience of 5 years or less. Of all participants, 86.4% reported that they or any of their family members had not been diagnosed with celiac disease. Of them, 21.8% had not heard about celiac disease and were not allowed to complete the questionnaire.

### Knowledge and attitude towards celiac disease

**Fig. 2:**
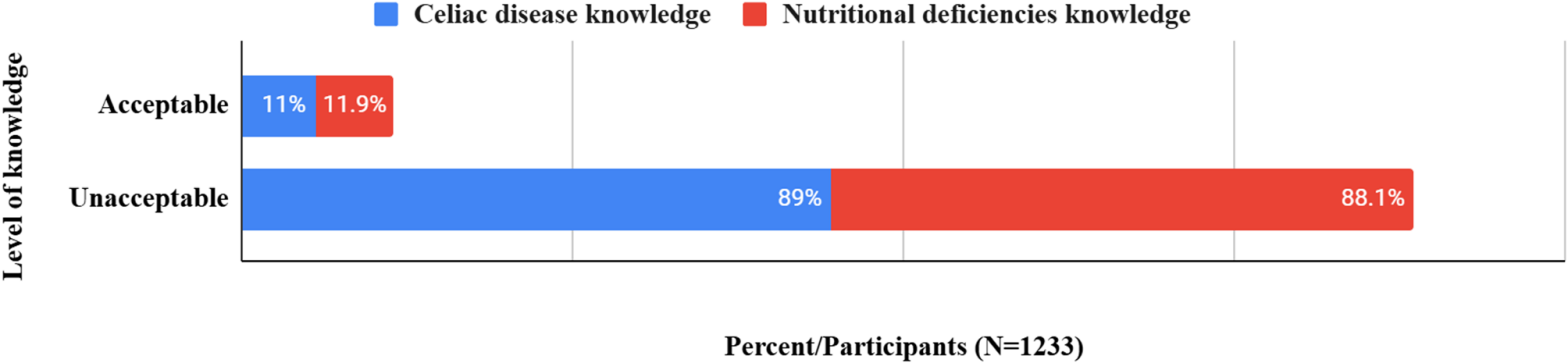
Levels of knowledge regarding celiac disease and its associated nutritional deficiencies among participants.

Figure 2 shows the prevalence of acceptable and unacceptable levels of knowledge. Only 11% of participants showed acceptable knowledge of celiac disease, with a median knowledge of 13 (8–19). The distribution of knowledge, correct answers, and attitudes towards celiac disease are shown in Table 2. 56.8% had the desire to know more about diagnostic and treatment methods, 51.5% about the symptoms, and 48.1% about the causes of the disease.

As shown in Table 4, women and those from urban residences had a higher median of knowledge, *p* = 0.003 and < = 0.001, respectively. Among the healthcare providers, the median of knowledge differed significantly according to specialty (*p* = 0.01), being higher in internal medicine physicians than pediatricians (*p* = 0.01) (Table 5). The place of work also had a significant effect (*p* = 0.01). With no multicollinearity and an adjusted R² of 0.07, backward linear regression revealed that urban residence and Egyptian nationality were predictors of high knowledge scores (β = 0.13, *p* = 0.05, 95% CI = -6.81 - -2.37) and (β = 0.27, *p* < 0.001, 95% CI = -7.13 - -0.04), respectively, with a large effect size (0.39).

### Knowledge and attitude towards gluten-free diets and their associated nutritional deficiencies

Only 11.9% of participants showed acceptable knowledge of the gluten-free diet and its associated nutritional deficiencies (Fig. 2). Distributions of knowledge, correct answers, and attitudes towards gluten-free diets and their associated nutritional deficiencies are shown in Table 3. (23.6%) thought that gluten-free food is widely available, and 20.6% thought that this diet can be managed without a dietitian’s help. Women and non-Egyptian participants had a higher median of knowledge, *p* = 0.05 and 0.03, respectively (Table 4). The median of knowledge differed significantly across regions of governorates (*p* = 0.002), with Delta governorates having a higher median than Greater Cairo (*p* = 0.004). The economic status also had a significant effect (*p* = 0.03), with a higher median at high economic levels compared to low levels (*p* = 0.05). With no multicollinearity and an adjusted R² of 0.05, backward linear regression revealed that older age groups were predictors of high knowledge scores (β = 0.22, *p* = 0.001, 95% CI = 0.54–2.22), with a small effect size (0.05). Scores of knowledge about the gluten-free diet and its associated nutritional deficiencies were positively correlated with scores of knowledge about celiac disease (*r* = 0.5, p < = 0.001).

**Table 1:** Socio-demographics of participants (*N* = 1233).

	*N*	%
Gender		
Men	734	59.5%
Women	499	40.5%
Age group (Years)		
< 30	1175	95.3%
30–40	29	2.4%
41–50	23	1.9%
> 50	6	0.5%
Nationality		
Egyptian	1089	88.3%
Non-Egyptian	144	11.7%
Governorate regions		
Greater Cairo	220	17.8%
Delta	895	72.6%
Coastal	58	4.7%
Frontiers	6	0.5%
Upper Egypt	54	4.4%
Residence		
Urban	859	69.7%
Rural	374	30.3%
Marital status		
Single	1139	92.4%
Not single	94	7.6%
Economic status		
High-level	172	13.9%
Average	984	79.8%
Low-level	77	6.2%
Academic level		
Pre-clinical student	158	12.8%
Clinical student	750	60.8%
A healthcare provider	325	26.4%
Healthcare providers (*N* = 325)		
Please indicate your place of work		
City Hospital	174	53.5%
District Hospital	57	17.5%
Private outpatient Hospital	24	7.4%
Research Center Hospital	19	5.8%
Republican hospital	20	6.2%
City outpatient public clinic	12	3.7%
Provincial Hospital	11	3.4%
Diagnostic outpatient center	8	2.5%
Please indicate your specialty		
Family Medicine	164	50.5%
Internal Medicine	84	25.8%
Pediatrics	62	19.1%
Intern	15	4.6%
Please indicate your work experience in the specialty		
Up to 5 years	241	74.2%
5–15 years	41	12.6%
More than 15 years	43	13.2%
Have you or any of your family members been diagnosed with celiac disease? (*N* = 1233)		
Yes	168	13.6%
No	1065	86.4%
Have you ever heard about celiac disease? (*N* = 1065)		
Yes	833	78.2%
No	232	21.8%

**Table 2:** Distributions of knowledge, correct answers, and attitudes towards Celiac disease (*N* = 833).

	*N*	%
What is celiac disease?		
Autoimmune	544	65.30
What causes celiac disease?		
Gluten intolerance	733	88
What symptoms and signs can you suspect of celiac disease in an adult (tick all that apply)?		
Chronic diarrhea or constipation	595	71.4
Weight deficiency	415	49.8
Iron deficiency anemia for an unknown reason	352	42.3
Frequent abdominal pain	579	69.5
Short stature	103	12.4
Osteoporosis	156	18.7
Irritable bowel syndrome	346	41.5
Chronic fatigue syndrome	282	33.9
Elevated hepatic enzymes for unknown reasons	108	13
No apparent symptoms	46	5.5
For what symptoms and signs can you suspect the presence of celiac disease in a child (tick all that apply)?*		
Chronic diarrhea or constipation	524	63.2
Frequent abdominal pain	506	61
Big belly	308	37.2
Vomiting	331	39.9
Weight deficiency, decreased muscle mass	456	55
Poor appetite	333	40.2
Short stature	286	34.5
Irritability, tearfulness	254	30.6
Iron deficiency anemia for an unknown reason	296	35.7
Frequent colds	87	10.5
Sometimes, no apparent symptoms	127	15.3
Which diseases can be associated with celiac disease (tick all that apply)?*		
Delayed sexual development in children	143	17.2
Infertility	129	15.5
Osteopenia, osteoporosis	248	29.8
Immunoglobulin A deficiency	230	27.6
Hypoplasia of tooth enamel	160	19.2
Recurrent aphthous stomatitis	150	18
Type A diabetes	280	33.6
Autoimmune thyroiditis	307	36.9
Autoimmune gastritis (pernicious anemia)	325	39
Herpetiformis dermatitis, psoriasis	157	18.8
Down syndrome, Turner syndrome	107	12.8
Peripheral neuropathy	77	9.2
What examination is necessary to confirm the diagnosis of celiac disease (the gold standard)?		
Fibrogastroduodenoscopy with small intestinal biopsy	228	27.4
What examination do you ask for if you suspect celiac disease in a patient (tick all that apply)?*^a^		
Fibrogastroduodenoscopy with small intestinal biopsy	292	35.1
Blood test for antibodies to tissue transglutaminase	359	43.1
Blood test for antibodies to endomysium	202	24.2
Blood test for antibodies to gliadin	276	33.1
None; I immediately refer to a gastroenterologist	39	4.7
Do you advise close relatives of patients with celiac disease to be examined for celiac disease?		
Yes	567	68.1
What is the main treatment for celiac disease?		
Lifetime Gluten-free diet	617	74.1
Would you like to know more about celiac disease? If so, what information would you like to receive? (Check all that apply)*		
I do not need to; I know enough	128	15.4
About diagnostic methods	473	56.8
About the treatment methods	473	56.8
About the causes of the disease	401	48.1
About the Symptoms	429	51.5

^a^No correct answers to this question.

*Multiple response analysis.

**Table 3:** Distributions of knowledge of correct answers and attitudes towards gluten-free diet and its associated nutritional deficiencies (*N* = 833).

	*N*	%
Do you think patients with celiac disease should follow a gluten-free diet that contains more calories compared to healthy people?		
No	279	33.5
In your opinion, can patients with celiac disease be exposed to nutritional deficiencies by following a gluten-free diet?		
Yes	596	71.5
Do you think individuals with celiac disease who follow a gluten-free diet can become overweight or obese?		
Yes	170	13.8
Do you think a gluten-free diet favors eating fewer complex carbohydrates?		
Yes	387	31.4
Do you think gluten-free processed foods contain more saturated fat than their gluten-containing counterparts?		
No	178	21.1
Do you think gluten-free processed foods contain more dietary fiber than their gluten-containing counterparts?		
Yes	313	25.4
Which micronutrient deficiencies may occur in patients with celiac disease as a result of following a gluten-free diet? (Choose all that apply)*		
Iron	398	47.8
Calcium	254	30.5
Zinc	239	28.7
Magnesium	211	25.3
Copper	178	21.4
Iodine	82	9.8
Which macronutrient deficiencies may occur in patients with celiac disease as a result of following a gluten-free diet? (Choose all that apply)*		
Vitamin A	261	31.3
Vitamin E	140	16.8
Vitamin D	267	32.1
Vitamin B1	148	17.8
Vitamin B12	281	33.7
Folic acid	227	27.3
Patients with celiac disease should be advised to do which of the following? (Choose all that apply)*		
Supplementation with multivitamin supplements	498	59.8
Vitamin D supplementation	284	34.1
Consuming products enriched with micronutrients	357	42.9
Do you think it is easy for celiac disease patients to acquire gluten-free food? Is it widely available?		
Strongly agree	38	4.6
Agree	158	19
Neutral	278	33.4
Disagree	266	31.9
Strongly disagree	93	11.2
To what extent do you think a patient with celiac disease can manage a gluten-free diet without a dietitian’s help due to the wide availability of gluten-free products?		
Strongly agree	37	4.4
Agree	135	16.2
Neutral	300	36
Disagree	283	34
Strongly disagree	78	9.4

*Multiple response analysis.

**Table 4:** Associations between sociodemographics and levels of knowledge.

	CD knowledge/35 (*N* = 829)		*p*-value	ND knowledge /21 (*N* = 833)		*p*-value
	Median	IQR		Median	IQR	
Gender			0.003* ^a^			0.05* ^**a**^
Men	13	7.75–18		6	3–10	
Women	14	9–20		7	4–10	
Age group			0.19^b^			0.09^b^
< 30	13	8–19		6	3–10	
30–40	13	10.25–24		7.5	4–10	
41–50	15.5	11.5–27.75		9	5–11.75	
> 50	16	7.5–18.5		10	6–14.75	
Nationality			0.58^a^			0.03* ^**a**^
Egyptian	13	8–19		6	3–10	
Non-Egyptian	12	9–17		7	4–11.5	
Residence			< 0.001* ^a^			0.69^a^
Urban	14	9–19		6	4–10	
Rural	12	7–17		6	3–10	
Marital status			0.08^a^			0.3^a^
Single	13	8–10		6	3–10	
Not single	14.5	10–20		6.5	4–10	
Governorate regions			0.3^b^			0.002* ^**b**^
Greater Cairo	12	7–17		5	2.5–8	0.004* ^**c **^
Delta	14	8.75–19		7	4–10	
Coastal	14	7.5–20		5	2.5–9.5	
Upper Egypt	14	7–20		6	4–12	
Economic status			0.06^b^			0.03* ^**b**^
High-level	15	10–20		7.5	4–11	0.05* ^**d**^
Average	13	8–19		6	3–10	
Low-level	12	5.5–17		5	3–7.5	

*CD* Celiac disease, *ND* Nutritional deficiency.

*Significant: ^a^Mann–Whitney U test, ^b^Kruska–Wallis H test.

^c^Adjusted significant association between Greater Cairo and Delta by pairwise comparison.

^d^Adjusted the significant association between High and Low levels by pairwise comparison.

**Table 5:** Difference in knowledge levels between students and healthcare providers.

	CD knowledge/35 (*N* = 829)		*p*-value	ND knowledge/21 (*N* = 833)		*p*-value
	Median	IQR		Median	IQR	
Academic level			0.15			0.5
Pre-clinical student	12	4.75–16.5		5	3–10	
Clinical student	13	8–19		6	3.25–10	
A healthcare provider	14	9–19		6.5	4–10	
For healthcare providers						
Please indicate your specialty			0.01*			0.02*
Family Medicine	14	9–18		5	3–9	0.03* ^a^
Internal Medicine	15	11–22		8	4–11	
Pediatrics	11	5–14	0.01* ^a^	6	3.5–9	
Intern	18	8.5–22.5		8	5.5–10.5	
Please indicate your work experience in the specialty			0.33			0.06
Up to 5 years	13.5	9–19		6	3–10	
5–15 years	15	9–23		6	4–9.5	
More than 15 years	15.5	11–21.25		9	6–12	
Please indicate your place of work			0.01*			0.12
City Hospital	15	10–21		7	3–10	
District Hospital	12	7–14		6	4–10	
Private outpatient Hospital	8	3.5–14.75		3	4–9	
Research center hospital	17	7–21.5		6	1.25–8.5	
Republican hospital	15	12.5–19		8.5	4.5–11	
City outpatient public clinic	11	6–14		4	5.25–11	
Provincial Hospital	22	9.5–26.5		9	1–6	
Diagnostic outpatient center	12	4–19.25		5	3.5–10	

*CD* Celiac disease, *ND* Nutritional deficiency.

*Significant.

All p-values are extracted from the Kruskal-Wallis H test.

^a^. Adjusted for significant association with internal medicine.

## Discussion

Celiac disease is a condition in which autoimmune cells attack the small intestine, triggered by the consumption of a gluten-containing diet in genetically predisposed individuals^1,2^. Although nutrition is an important factor in the treatment of CD and its associated nutritional deficiencies, this lack of knowledge leads to misdiagnosis and improper dietary recommendations^10,11^. The prevalence of celiac disease among Egyptian children is about 0.53%, making it a growing public health concern^14^. However, data on healthcare professionals and medical students about the awareness of celiac diseases and related nutritional deficiencies is limited. Focusing on improving education and training for future healthcare professionals can resolve this knowledge gap^12,13,16^. This study highlighted a significant lack of knowledge regarding celiac disease and its associated nutritional deficiency among Egyptian medical students and healthcare professionals. This knowledge gap could contribute to misdiagnosis and inadequate nutritional counseling.

The study also revealed several misconceptions regarding celiac disease, particularly about its symptoms and diagnostic methods. For example, only 18.7% of participants knew osteoporosis is a symptom of celiac disease, and just 27.4% identified intestinal biopsy as the gold standard method for diagnosis. Such gaps in awareness may delay diagnosis, resulting in extended discomfort for the patient and an increased risk of consequences. These data suggest that CD is frequently disregarded or misdiagnosed, potentially leading to delayed treatment and increased consequences such as malnutrition, anemia, and osteoporosis^5,14,17^. While 71.5% acknowledged potential nutritional deficiency, fewer were able to correctly identify specific deficiencies. This gap in awareness of this important nutritional deficiency is crucial. For instance, improper dietary management will exacerbate malnutrition in celiac disease patients. However, there was one hopeful sign: 59.8% of participants suggested multivitamin supplementation, indicating some understanding of the importance of nutritional support.

Regarding knowledge of celiac disease, certain demographic factors, such as urban residents, are showing a higher level of awareness. Urban populations may have superior healthcare facilities and access to medical education resources, which could explain their higher ratings^18^. Also, Egyptian women show a higher level of awareness. This aligns with studies suggesting that women tend to be more engaged in dietary and nutrition-related topics^19,20^. The observed result of the study aligns with the previous research by identifying significant knowledge gaps among healthcare professionals and medical students regarding celiac disease and its management. Only 11% of participants showed an acceptable level of knowledge, consistent with previous findings that highlighted the lack of awareness about celiac disease symptoms, diagnostic methods, and dietary recommendations^21,22^. These findings contrast with those in the Saudi Arabian study, which revealed adequate knowledge regarding celiac disease^23^. Similar to global studies, this research revealed a limited understanding of nutritional deficiencies associated with a gluten-free diet, such as deficiencies in iron, vitamin D, and vitamin B12, with only 11.9% of participants demonstrating acceptable knowledge and 59.8% of participants recommending multivitamin supplementation for celiac patients^24–26^. Predictors of high knowledge, such as urban residents, high socioeconomic status, and female gender, were in line with prior studies^27,28^. Additionally, the expressed desire for more education about celiac disease and its treatment was reported by 56.8% of participants.

In alignment with those studies^29,30^, the results were also unsatisfactory when evaluating knowledge of the gluten-free diet and its associated nutritional influence on celiac disease. Suggesting that celiac disease and its dietary management are not adequately emphasized in medical training^29,30^. This deficiency in dietary knowledge is particularly concerning, as improper adherence to the GFD leads to nutritional imbalances and worsening patient health outcomes^31,32^. Several demographic factors influence knowledge levels. Older participants and those from higher economic backgrounds showed a greater level of awareness, likely due to greater opportunities for continuing medical education and access to updated medical resources^27,28^. Furthermore, non-Egyptian women had better knowledge of GFD, possibly due to potential differences in exposure to information or educational curriculum. What also needs to be highlighted is the strong correlation between knowledge of celiac disease and knowledge of gluten-free diets (*r* = 0.5, *p* ≤ 0.001), suggesting that improving education about celiac disease can enhance the understanding of the recommended dietary management as well^33^. This reinforces the need for a comprehensive educational approach that encompasses both disease pathology and nutritional aspects, ensuring that future healthcare providers are well-educated to improve patient outcomes.

This research emphasizes the critical necessity for nutritional education in medical education. This will enable future medical professionals to assist patients with celiac disease with the right dietary advice. Regarding gluten-free diet-related deficiency in celiac disease patients, only 23.6% of participants believed that gluten-free diets are widely available. This contrasts with findings from developing countries, where healthcare providers often consider gluten-free products more accessible^34^. This disparity is likely the result of several Egypt-specific factors. To begin with, Egypt lacks comprehensive food labeling laws, and it is difficult for consumers to identify gluten-free products short of importing them, which limits availability. Secondly, while there are a few specialty grocery stores and gluten-free dedicated bakeries in urban areas like Cairo and Alexandria, they are limited in number and tend to cater to niche markets, with much of the population underserved. Besides, cultural reliance on wheat staples like bread exacerbates challenges in adopting gluten-free diets. By contrast, countries like Saudi Arabia have witnessed improved availability of gluten-free products due to increased awareness and market demand^35^. Addressing these disparities through better labeling regulations and expanding access to affordable gluten-free products could improve attitudes and adherence to gluten-free diets in Egypt. The study also indicates that 20.6% of participants thought a gluten-free diet could be managed without dietitians, differing from prior studies that underscore the crucial role of dietitians in Celiac disease management^36,37^. These findings, while consistent with prior research on general knowledge gaps and predictors of awareness, highlight important contextual differences in training, dietary practices, and healthcare systems, emphasizing the need for targeted interventions to address these gaps and improve education and awareness in Egypt.

However, the study diverged from past research in some aspects, notably finding that internal medicine physicians had greater knowledge than pediatricians. This contrasts with other studies, which typically reported pediatricians as more knowledgeable due to their often extensive exposure to celiac disease in childhood cases^38,39^. This may be explained by their greater diagnostic role for adults, where CD presents non-classical symptoms (e.g., anemia, osteoporosis) requiring greater knowledge in terms of long-term management and nutritional deficiencies. Internal medicine physicians routinely request testing and referrals, driving knowledge acquisition, whereas pediatricians may rely on specialists or multidisciplinary teams for the management of CD, rendering broad expertise less requisite for them. Also, ongoing medical education programs can accord more weight to CD in internal medicine doctors, who must contend with previously undiagnosed cases in the adult population, than in pediatricians specializing in developmental or acute disorders. That disparity warrants specialized training to apprise pediatricians of CD’s atypical manifestations and nutritional implications, particularly in Egypt and other countries with high prevalence and demonstrated disparities in care.

### Specific proposals or strategies

Focused education needs to be promoted to increase knowledge and attitudes among healthcare professionals regarding celiac disease. Including multidisciplinary modules on the management of celiac disease and nutrition in undergraduate and postgraduate medical curricula can lead to the early development of core knowledge. In addition to this, the availability of continuing medical education (CME) workshops, seminars, and online courses provides practicing clinicians with an opportunity to update their knowledge regarding diagnosis, treatment, and patient management^40^. Multidisciplinary education programs of dietitians, gastroenterologists, and primary care physicians can facilitate interprofessional care practices, resulting in improved patient outcomes^41^. Besides this, the development and sharing of evidence-based educational resources relevant to local settings enables clinicians to prioritize giving appropriate nutritional guidance^42^. Finally, promoting patient-centered care with open communication and support networks empowers patients and healthcare providers to adhere to gluten-free diets and improve quality of life^43^. Such multifaceted interventions involving formal instruction, experiential learning, and patient engagement are necessary to close celiac disease knowledge gaps and improve celiac disease care globally.

### Limitations and recommendations

The study has several strong points, including a large sample size, which enhances the reliability and generalization of findings. Moreover, it includes both medical students and healthcare professionals from various specialties, ensuring a comprehensive assessment across experience levels. The research addresses an unexplored area, as there is limited data on celiac disease awareness among Egyptian medical students, making it a significant contribution. It effectively identifies key knowledge gaps, particularly in symptoms, diagnostic procedures, and nutritional management, provided by these clear targets for educational improvement. Additionally, it examines attitudes and practices, revealing misconceptions among future healthcare providers. The use of rigorous statistical analyses (Mann-Whitney U test, Kruskal-Wallis test, regression analysis) ensures accurate data interpretation and bolsters the validity of the conclusions. By exploring the relationship between CD knowledge and awareness of a gluten-free diet, the study enhances the understanding of how training in one area affects another. Its findings have practical implications for curriculum development, guiding efforts to better prepare healthcare providers for diagnosing and managing CD. Furthermore, the study adheres to ethical guidelines, received institutional approval, and conducted a pilot test to refine the questionnaire, thus improving the quality of data collection. Lastly, it identifies demographic and socioeconomic predictors of knowledge, helping tailor educational interventions to specific groups. These strengths make the study a valuable resource for enhancing CD education and awareness among medical students and healthcare professionals in Egypt. Despite its valuable insights, this study has some limitations. The use of convenience sampling may introduce selection bias, and the reliance on self-reported knowledge could lead to over- or underestimation of actual competency levels. Additionally, the study primarily focused on medical students and HCPs in Egypt, limiting the generalizability of findings to other regions. We recommend stratified sampling in future studies to ensure representation from rural and underrepresented governorates. The study’s self-reported knowledge has social desirability bias (overestimation of capacity to live up to professional demands by participants) and recall bias (self-perceived inaccuracy due to lack of memory or lack of exposure in the clinic) threats. Though the paper makes mention of a push towards voluntary participation, strict mitigation procedures (e.g., anonymity assurance, triangulation using objective scales) were not made. Subsequent research should address these limitations by including case-based assessments (e.g., clinical vignette testing applied knowledge) or objective tests to validate self-reported claims, such that results reflect real-world competency rather than self-perceived expertise. Such an addition would improve validity, particularly in Egypt’s context of elevated CD prevalence and documented deficits in reported care, where objective measurement might identify significant discrepancies between self-reporting and clinical practice.

## Conclusion

In summary, this study highlights the limited awareness of CD knowledge and the related nutritional deficiencies among Egyptian medical students and healthcare providers, particularly concerning symptoms, diagnostic methods, and dietary management. Given the increasing prevalence of CD in Egypt, addressing these knowledge gaps is essential to improving early diagnosis and patient care. Targeted educational initiatives, interdisciplinary collaboration, and public awareness campaigns are critical to ensuring that future HCPs are well-equipped to manage CD effectively.

## Electronic supplementary material

Below is the link to the electronic supplementary material.

Supplementary Material 1

## Data Availability

The relevant authors of the current study have kindly provided the data sets under analysis upon appropriate demand from the corresponding author.

## References

[1] Al Ibrahmi, B. & Bour, A. A short update on new approaches to Celiac disease. *Acta Biomed.* **93** (6), e2022322. 10.23750/ABM.v93i6.13673 (2022). 36533746 10.23750/abm.v93i6.13673 PMC9828896

[2] Brietzke, E., Cerqueira, R. O., Mansur, R. B. & McIntyre, R. S. Gluten-related illnesses and severe mental disorders: a comprehensive review. *Neurosci. Biobehav Rev.* **84**, 368–375. 10.1016/j.neubiorev.2017.08.009 (2018). 28830676 10.1016/j.neubiorev.2017.08.009

[3] Catassi, C., Verdu, E. F., Bai, J. C. & Lionetti, E. Coeliac disease. *Lancet* **399** (10344), 2413–2426. 10.1016/S0140-6736(22)00794-2 (2022). 35691302 10.1016/S0140-6736(22)00794-2

[4] Anderson, R. P. Innate and adaptive immunity in Celiac disease. *Curr. Opin. Gastroenterol.* **36** (6), 470–478. 10.1097/MOG.0000000000000672 (2020). 32889822 10.1097/MOG.0000000000000672

[5] Al-Toma, A. et al. European society for the study of coeliac disease (ESsCD) guideline for coeliac disease and other gluten-related disorders. *United Eur. Gastroenterol. J.* **7** (5), 583–613. 10.1177/2050640619844125 (2019). 10.1177/2050640619844125 PMC6545713 31210940

[6] Daley, S. F., Posner, E. B. & Haseeb, M. Celiac disease. In *StatPearls* (StatPearls Publishing, 2025). 28722929

[7] Durazzo, M. et al. Extra-Intestinal manifestations of Celiac disease: what should we know in 2022? *J. Clin. Med.* **11** (1), 258. 10.3390/jcm11010258 (2022). 35011999 10.3390/jcm11010258 PMC8746138

[8] Assiri, A. M. et al. Assessment of knowledge of Celiac disease among health care professionals. *Saudi Med. J.* **36** (6), 751–753. 10.15537/smj.2015.6.11519 (2015). 25987121 10.15537/smj.2015.6.11519 PMC4454913

[9] Sahin, Y. et al. Knowledge regarding Celiac disease among healthcare professionals, patients, and their caregivers in Turkey. *World J. Gastrointest. Pathophysiol*. **13** (6), 178–185. 10.4291/wjgp.v13.i6.178 (2022). 36532302 10.4291/wjgp.v13.i6.178 PMC9752282

[10] Dembiński, Ł., Mazur, A., Dąbrowski, M., Jackowska, T. & Banaszkiewicz, A. Knowledge of medical students and medical professionals regarding nutritional deficiencies in patients with Celiac disease. *Nutrients* **13** (6), 1771. 10.3390/nu13061771 (2021). 34067382 10.3390/nu13061771 PMC8224609

[11] Ageely, H. et al. Knowledge of health students regarding nutritional deficiencies in patients with Celiac disease in Jazan region: A Cross-Sectional study. *Cureus* **16** (6), e62558 (2024). 39027792 10.7759/cureus.62558 PMC11254512

[12] Barzegar, F. et al. Lack of healthcare professionals’ awareness of the management of Celiac disease May contribute to the underdiagnosis of Celiac disease. *Gastroenterol. Hepatol. Bed Bench*. **12** (3), 203–208 (2019). 31528303 PMC6668761

[13] Kozhakhmetova, A., Aidossov, S., Kapassova, A. & Borsoldayeva, K. Current knowledge and Myths about Celiac disease among physicians in the Republic of kazakhstan: A countrywide cross-sectional study. *Front. Public. Health*. **10**, 956135. 10.3389/fpubh.2022.956135 (2022). 36033766 10.3389/fpubh.2022.956135 PMC9411637

[14] Abu-Zekry, M., Kryszak, D., Diab, M., Catassi, C. & Fasano, A. Prevalence of Celiac disease in Egyptian children disputes the east-west agriculture-dependent spread of the disease. *J. Pediatr. Gastroenterol. Nutr.* **47** (2), 136–140. 10.1097/MPG.0b013e31815ce5d1 (2008). 18664863 10.1097/MPG.0b013e31815ce5d1

[15] Dina, I. S., Safaa, T. & Heba, S. A. Celiac disease and adherence to a gluten-free diet: an outpatient view. *Bull. Natl. Nutr. Inst. Arab. Repub. Egypt.* **50** (1), 1–20. 10.21608/bnni.2017.6721 (2017).

[16] Zipser, R. D., Farid, M., Baisch, D., Patel, B. & Patel, D. Physician awareness of Celiac disease: a need for further education. *J. Gen. Intern. Med.* **20** (7), 644–646. 10.1111/j.1525-1497.2005.0107.x (2005). 16050861 10.1111/j.1525-1497.2005.0107.x PMC1490146

[17] Bai, J. C. et al. World gastroenterology organisation global guidelines on Celiac disease. *J. Clin. Gastroenterol.* **47** (2), 121–126. 10.1097/MCG.0b013e31827a6f83 (2013). 23314668 10.1097/MCG.0b013e31827a6f83

[18] Chen, X. et al. Differences in rural and urban health information access and use. *J. Rural Health*. **35** (3), 405–417. 10.1111/jrh.12335 (2019). 30444935 10.1111/jrh.12335 PMC6522336

[19] Kinyua, L. W. *Association of Nutrition Knowledge and Attitude with Dietary Practices and Nutritional Status of Female Undergraduate Students Attending University Colleges Within Nairobi Metropolis [doctoral dissertation]*. University of Nairobi (2013).

[20] Alotaibi, N. M. et al. A cross-sectional study of gender differences in calorie labeling policy among students: dietary habits, nutritional knowledge and awareness. *Nutrients* **15** (4), 879. 10.3390/nu15040879 (2023). 36839237 10.3390/nu15040879 PMC9958862

[21] Hall, S., Kenrick, K., Day, A. S. & Vernon-Roberts, A. A systematic review of tools to assess coeliac disease-related knowledge. *J. Clin. Med.* **13** (14), 4053. 10.3390/jcm13144053 (2024). 10.3390/jcm13144053 PMC11277601 39064096

[22] Riznik, P. et al. Uncovering the gap: coeliac disease knowledge among healthcare professionals in the Danube region. *BMC Gastroenterol.* **24** (1), 254. 10.1186/s12876-024-03349-x (2024). 39123100 10.1186/s12876-024-03349-x PMC11312701

[23] Alhuzaim, W. M. et al. Knowledge and attitude of Celiac disease among the population of riyadh, Saudi Arabia. *Cureus* **16** (9), e68603. 10.7759/cureus.68603 (2024). 39371695 10.7759/cureus.68603 PMC11450511

[24] Vici, G., Belli, L., Biondi, M. & Polzonetti, V. Gluten-free diet and nutrient deficiencies: A review. *Clin. Nutr.* **35** (6), 1236–1241. 10.1016/j.clnu.2016.05.002 (2016). 27211234 10.1016/j.clnu.2016.05.002

[25] Dennis, M., Lee, A. R. & McCarthy, T. Nutritional considerations of the Gluten-Free diet. *Gastroenterol. Clin. North. Am.* **48** (1), 53–72. 10.1016/j.gtc.2018.09.002 (2019). 30711211 10.1016/j.gtc.2018.09.002

[26] Di Nardo, G. et al. Nutritional deficiencies in children with celiac disease resulting from a gluten-free diet: A systematic review. *Nutrients* **11** (7), 1588. 10.3390/nu11071588 (2019). 10.3390/nu11071588 PMC6683263 31337023

[27] Peters, D. H. et al. Poverty and access to health care in developing countries. *Ann. N Y Acad. Sci.* **1136**, 161–171. 10.1196/annals.1425.011 (2008). 17954679 10.1196/annals.1425.011

[28] Richardson, A., Allen, J. A., Xiao, H. & Vallone, D. Effects of race/ethnicity and socioeconomic status on health information-seeking, confidence, and trust. *J. Health Care Poor Underserved*. **23** (4), 1477–1493. 10.1353/hpu.2012.0181 (2012). 23698662 10.1353/hpu.2012.0181

[29] Crespo-Escobar, P. et al. Knowledge gaps in Gluten-Free diet awareness among patients and healthcare professionals: A call for enhanced nutritional education. *Nutrients* **16** (15), 2512. 10.3390/nu16152512 (2024). 39125392 10.3390/nu16152512 PMC11314127

[30] Herrera-Quintana, L. et al. Celiac disease: beyond diet and food awareness. *Foods* **14** (3), 377. 10.3390/foods14030377 (2025). Published 2025 Jan 24. 39941971 10.3390/foods14030377 PMC11817883

[31] Bianchi, P. I. et al. Nutritional consequences of Celiac disease and Gluten-Free diet. *Gastroenterol. Insights*. **15** (4), 878–894. 10.3390/gastroent15040061 (2024).

[32] Simón, E. et al. The Gluten-Free diet for Celiac disease: critical insights to better understand clinical outcomes. *Nutrients* **15** (18), 4013. 10.3390/nu15184013 (2023). 37764795 10.3390/nu15184013 PMC10537989

[33] Cazzetta, S. et al. Educational needs in the diagnosis and management of patients with Celiac disease: a practice management survey of healthcare professionals. *Am. J. Gastroenterol.* **119** (10S), S1604–S1605. 10.14309/01.ajg.0001038352.03390.b4 (2024).

[34] Hanci, O. & Jeanes, Y. M. Are gluten-free food staples accessible to all patients with coeliac disease? *Frontline Gastroenterol.* **10** (3), 222–228. 10.1136/flgastro-2018-101088 (2019). 31281622 10.1136/flgastro-2018-101088 PMC6583765

[35] Qashqari, L., Shakweer, D., Alzaben, A. S. & Hanbazaza, M. A. Investigation of the cost and availability of gluten-free food in jeddah, KSA. *J. Taibah Univ. Med. Sci.* **19** (2), 422–428. 10.1016/j.jtumed.2024.02.001 (2024). 38419959 10.1016/j.jtumed.2024.02.001 PMC10899026

[36] Gładyś, K. et al. Expanded role of a dietitian in monitoring a Gluten-Free diet in patients with Celiac disease: implications for clinical practice. *Nutrients* **13** (6), 1859. 10.3390/nu13061859 (2021). 34072491 10.3390/nu13061859 PMC8228256

[37] Weiss, G. *Diagnosis and Management of Gluten-Associated Disorders: A Clinical Casebook*. 10.1007/978-3-030-56722-4 (Springer, 2021).

[38] Malik, I. et al. Celiac disease: what the Indian pediatricians know about the disease. *Indian J. Gastroenterol.* **38** (3), 263–267. 10.1007/s12664-019-00958-3 (2019). 31254168 10.1007/s12664-019-00958-3

[39] Vieira, C. et al. What do Brazilian pediatricians know about Celiac disease? *Dig. Dis. Sci.* **56** (3), 799–804. 10.1007/s10620-010-1339-6 (2011). 20632095 10.1007/s10620-010-1339-6

[40] Daley, S. F. & Haseeb, M. Celiac disease. In *StatPearls [Internet]*. https://www.ncbi.nlm.nih.gov/books/NBK441900/ (StatPearls Publishing, 2025). 28722929

[41] Eliot, K. A., L’Horset, A. M., Gibson, K. & Petrosky, S. Interprofessional education and collaborative practice in nutrition and dietetics 2020: an update. *J. Acad. Nutr. Diet.* **121** (4), 637–646 (2021). 33008786 10.1016/j.jand.2020.08.010

[42] Love, J. N., Messman, A. M. & Merritt, C. Improving the learning experience through Evidence-based education. *West. J. Emerg. Med.* **20** (1), 1–5. 10.5811/westjem.2018.10.41320 (2019). 30643592 10.5811/westjem.2018.10.41320 PMC6324697

[43] Fukami, T. Enhancing healthcare accountability for administrators: fostering transparency for patient safety and quality enhancement. *Cureus* **16** (8), e66007. 10.7759/cureus.66007 (2024). 39221336 10.7759/cureus.66007 PMC11366401

