# Supplementary material for: Knowledge and attitudes toward nutritional deficiencies in celiac disease among medical students and healthcare providers in Egypt
Source: Sci Rep. 2025 Jul 31;15:27894. doi: 10.1038/s41598-025-12247-5 (PMC12310944; doi:10.1038/s41598-025-12247-5)
Supplement: Supplementary file 1 — Supplementary Material 1 [file 41598_2025_12247_MOESM1_ESM.docx]

**Knowledge and Attitudes toward Nutritional Deficiencies in Celiac Disease among Medical Students and Healthcare Providers in Egypt**

Mohammed. N. Abdelaziz^1^, Hajer Azzam^1^, Abdalla Hefnawy^1^, AHMED. R. A. MOUSTAFA^1^, Elkasabi team^2^, Omar Abdallah^3^

1. Medical Intern, Faculty of Medicine, Mansoura University, Egypt.
2. Medical student, Manchester program, Faculty of Medicine, Mansoura University, Egypt.
3. Lecturer of Hepatology and Gastroenterology, Faculty of Medicine, Mansoura University, Egypt.

Correspondence: Mohammed. N. Abdelaziz: Medical Intern, Faculty of Medicine, Mansoura University, Egypt. Postal address: Arab Republic of Egypt, Al-Daqahlia Governorate, Mansoura, Mansoura. Postal office number 35516. E-mail: mohammednasser@std.mans.edu.eg ORCID: 0000-0003-2699-2901.

**Supplementary appendix 1:**

Celiac study validated questionnaire

**Supplementary appendix 2:**

STROBE checklist

**SUPPLEMENTARY APPENDIX 1**

**Questionnaire on Celiac Disease**

**Section 1:- demographic data**

**1. Please indicate your gender.**

☐ Male

☐ Female

**2. Please indicate your age group.**

☐ Under 30

☐ 30-40 years

☐ 40-50 years

☐ Over 50

**3. Nationality:**

☐ Egyptian Arab

☐ Other (Please specify) ___

4. **Governorate of Residence in Egypt**:

☐ Dakahlia

☐ Other (Please specify) ___

**5. For students, please indicate the Year of Study in Medical School:**

☐ 1st Year

☐ 2nd Year

☐ 3rd Year

☐ 4th Year

☐ 5th Year

**6. For healthcare providers, Please indicate your place of work.**

☐ District hospital

☐ City hospital

☐ Province hospital

☐ Republican hospital

☐ City out-patient public clinic

☐ Diagnostic out-patient center

☐ Research Center hospital

☐ Private out-patient clinic

☐Other______

**7. For healthcare providers, please indicate your specialty.**

☐ General practitioner

☐ Pediatrics

☐ Internal medicine

☐ Other___________

**8. For healthcare providers, Please indicate your work experience in the specialty.**

☐ Up to 5 years

☐ 5-15 years

☐ More than 15 years

**9. Residence:**

☐ Urban

☐ Rural

**10. Marital status:-**

☐ Single

☐ Married

**11. Economic status:-**

☐ High level

☐ Average

☐ Low level

**12- Have you or any of your family members been diagnosed with celiac disease?**

☐ Yes

☐ No

**13- Have you ever heard about celiac disease?**

☐ Yes

☐ No

**Section 2 knowledge and attitude regarding celiac disease**

**14. What is celiac disease?**

☐ Allergic disease

☐ Autoimmune disease

☐ Infectious disease

☐ Large bowel disorder

☐ Genetic disorder, gene mutation leads to disease in 100% of mutation carriers

☐ I don’t know

**15. What causes celiac disease?**

☐ Gluten intolerance

☐ Intolerance to dairy products

☐ Allergy

☐ Gut dysbiosis

☐ I don't know

**16. What symptoms and signs can you suspect celiac disease in an adult (tick all that apply)?**

☐ Adults do not have celiac disease, it is a childhood disease

☐ Chronic diarrhea or constipation

☐ Weight deficiency

☐ Iron deficiency anemia for unknown reasons

☐ Frequent abdominal pain and bloating

☐ Short stature

☐ Osteoporosis

☐ Presence of irritable bowel syndrome

☐ The presence of chronic fatigue syndrome

☐ Elevated hepatic ALT and AST for unknown reasons

☐ No apparent symptoms.

☐ I do not know, I am a pediatrician

☐ I don't know

**17. For what symptoms and signs can you suspect the presence of celiac disease in a child (tick all that apply)?**

☐ Chronic diarrhea or constipation

☐ Frequent abdominal pain

☐ Big belly

☐ Vomiting

☐ Weight deficiency, decreased muscle mass

☐ Poor appetite

☐ Short stature

☐ Irritability, tearfulness

☐ Iron deficiency anemia for unknown reasons

☐ Frequent colds

☐ Sometimes no apparent symptoms.

☐ I do not know, I only treat adults

☐ I don't know

**18. Which of the following diseases can be associated with celiac disease (tick all that apply)?**

☐ Delayed sexual development in children

☐ Infertility

☐ Osteopenia, osteoporosis

☐ Immunoglobulin A deficiency

☐ Hypoplasia of tooth enamel

☐ Recurrent aphthous stomatitis

☐ Type 1 diabetes

☐ Autoimmune thyroiditis

☐ Autoimmune gastritis (pernicious anemia)

☐ Herpetiformis dermatitis, psoriasis

☐ Down syndrome, Turner syndrome

☐ Peripheral neuropathy, ataxia, epilepsy

☐ I don't know

**19. What examination do you prescribe if you suspect celiac disease in a patient (tick all that apply)?**

☐ Stomach examination (plain fibrogastroduodenoscopy)

☐ Fibrogastroduodenoscopy with small intestinal biopsy

☐ Ultrasound of the pancreas

☐ Examination of feces for fat (coprogram)

☐ Blood test for antibodies to tissue transglutaminase

☐ Blood test for antibodies to endomysium

☐ Blood test for antibodies to gliadin

☐ Genotyping of HLA DQ2 and DQ8

☐ None, I advise patient to try a gluten-free diet for a while

☐ None, I immediately refer to endocrinologist

☐ None, I immediately refer to gastroenterologist

☐ I don't know

**20. What examination is necessary to confirm the diagnosis of celiac disease (golden standard)?**

☐ Antibodies to tissue transglutaminase

☐ Antibodies to endomysium

☐ Antibodies to gliadin

☐ Genotyping for HLA DQ2 and DQ8

☐ Fibrogastroduodenoscopy with small intestinal biopsy

☐ I don't know

**21. Do you advise close relatives of patients with celiac disease to be examined for celiac disease?**

☐ Yes

☐ No

**22. What is the main treatment for celiac disease?**

☐ Dairy-free diet

☐ Antihistamines

☐ Gluten-free diet for 1 month

☐ H. pylori eradication

☐ Lifetime gluten-free diet

**23. Would you like to know more about celiac disease? If so, what information would you like to receive?**

☐ About the causes of the disease

☐ About the Symptoms

☐ About diagnostic methods

☐ About the treatment methods

☐ I do not need, I know enough

☐ Other__________

**Section 3: knowledge regarding nutritional deficiencies related to celiac**

**24. Do you think patients with celiac disease should follow a gluten-free diet that contains more calories compared to healthy people?**

☐ Yes

☐ No

**25. In your opinion, Patients with celiac disease can be exposed to nutritional deficiencies by following a gluten-free diet.**

☐ Yes

☐ No

**26. In your opinion, deficiencies of which micronutrients may occur in patients with celiac disease following a gluten-free diet?**

☐ Iron

☐ calcium

☐ zinc

☐ magnesium

☐ copper

☐ iodine

☐ I don’t know

**27. Deficiencies of which vitamins can occur in patients with celiac disease following a gluten-free diet?**

☐ vitamin A

☐ vitamin E

☐ vitamin C

☐ vitamin D

☐ vitamin B1

☐ vitamin B12

☐ folic acid

☐ I don’t know

**28. Patients with celiac disease should be advised:**

☐ supplementation with multivitamin drugs

☐ vitamin D supplementation

☐ consuming products enriched with micronutrients

☐ a gluten-free diet alone is sufficient

☐ I don’t know

**29. To what extent do you think Individuals with celiac disease who follow a gluten-free diet can become overweight or obese?**

☐ Strongly agree

☐ Agree

☐ Neutral

☐ Disagree

☐ Strongly disagree

**30. To what extent do you think a gluten-free diet favors eating fewer complex carbohydrates?**

☐ Strongly agree

☐ Agree

☐ Neutral

☐ Disagree

☐ Strongly disagree

**31. To what extent do you think Gluten-free processed foods contain more saturated fat than their gluten-containing counterparts**?

☐ Strongly agree

☐ Agree

☐ Neutral

☐ Disagree

☐ Strongly disagree

**32. To what extent do you think Gluten-free processed foods contain more dietary fiber than their gluten-containing counterparts?**

☐ Strongly agree

☐ Agree

☐ Neutral

☐ Disagree

☐ Strongly disagree

**33. To what extent do you think all patients with celiac disease should have regular assessments of vitamin D levels, regardless of their supplementation?**

☐ Strongly agree

☐ Agree

☐ Neutral

☐ Disagree

☐ Strongly disagree

**34. To what extent do you think a gluten-free diet in a patient with celiac disease does not require a dietician's consultation due to the widespread access to gluten-free products?**

☐ Strongly agree

☐ Agree

☐ Neutral

☐ Disagree

☐ Strongly disagree

STROBE Statement—Checklist of items that should be included in reports of ***cross-sectional studies***

|  | Item No | Recommendation | Addressed in Manuscript |
| --- | --- | --- | --- |
| **Title and abstract** | 1 | (*a*) Indicate the study’s design with a commonly used term in the title or the abstract | Title, Abstract |
|  |  | (*b*) Provide in the abstract an informative and balanced summary of what was done and what was found | Abstract |
| Introduction | | |  |
| Background/rationale | 2 | Explain the scientific background and rationale for the investigation being reported | Introduction, Paragraphs 1-3 |
| Objectives | 3 | State-specific objectives, including any prespecified hypotheses | Introduction, Final paragraph |
| Methods | | |  |
| Study design | 4 | Present key elements of the study design early in the paper | Methods, “Population and study design” |
| Setting | 5 | Describe the setting, locations, and relevant dates, including periods of recruitment, exposure, follow-up, and data collection | Methods, “Population and study design” |
| Participants | 6 | (*a*) Give the eligibility criteria, and the sources and methods of selection of participants | Methods, “Population and study design” |
| Variables | 7 | Clearly define all outcomes, exposures, predictors, potential confounders, and effect modifiers. Give diagnostic criteria, if applicable | Methods, "Questionnaire Deployment" |
| Data sources/ measurement | 8* | For each variable of interest, give sources of data and details of methods of assessment (measurement). Describe the comparability of assessment methods if there is more than one group | Methods, “Questionnaire Deployment” |
| Bias | 9 | Describe any efforts to address potential sources of bias | Methods, "Population and study design" |
| Study size | 10 | Explain how the study size was arrived at | Methods, "Population and study design" |
| Quantitative variables | 11 | Explain how quantitative variables were handled in the analyses. If applicable, describe which groupings were chosen and why | Methods, "Statistical analysis" |
| Statistical methods | 12 | (*a*) Describe all statistical methods, including those used to control for confounding | Methods, "Statistical analysis" |
|  |  | (*b*) Describe any methods used to examine subgroups and interactions | Methods, "Statistical analysis" |
|  |  | (*c*) Explain how missing data were addressed | Methods, "Statistical analysis" |
|  |  | (*d*) If applicable, describe analytical methods taking account of sampling strategy | Methods, "Statistical analysis" |
|  |  | (*e*) Describe any sensitivity analyses | Not applicable |
| Results | | |  |
| Participants | 13* | (a) Report numbers of individuals at each stage of study—eg numbers potentially eligible, examined for eligibility, confirmed eligible, included in the study, completing follow-up, and analysed | Results, "Socio-demographic analysis"; Figure 1 (Flowchart) |
|  |  | (b) Give reasons for non-participation at each stage | Methods, "Population and study design"; Figure 1 (Flowchart) |
|  |  | (c) Consider use of a flow diagram | Methods, "Population and study design"; Figure 1 (Flowchart) |
| Descriptive data | 14* | (a) Give characteristics of study participants (eg demographic, clinical, social) and information on exposures and potential confounders | Results, "Socio-demographic analysis"; |
|  |  | (b) Indicate number of participants with missing data for each variable of interest | Results, "Socio-demographic analysis"; Figure 1 (Flowchart) |
| Outcome data | 15* | Report numbers of outcome events or summary measures | Results, Tables 2–5, Figure 1 |
| Main results | 16 | (*a*) Give unadjusted estimates and, if applicable, confounder-adjusted estimates and their precision (eg, 95% confidence interval). Make clear which confounders were adjusted for and why they were included | Results, Tables 4–5; Statistical analysis |
|  |  | (*b*) Report category boundaries when continuous variables were categorized | Results, Tables 4–5 |
|  |  | (*c*) If relevant, consider translating estimates of relative risk into absolute risk for a meaningful time period | Results |
| Other analyses | 17 | Report other analyses done—eg analyses of subgroups and interactions, and sensitivity analyses | Not applicable |
| Discussion | | |  |
| Key results | 18 | Summarise key results with reference to study objectives | Discussion, Paragraph 1 |
| Limitations | 19 | Discuss limitations of the study, taking into account sources of potential bias or imprecision. Discuss both direction and magnitude of any potential bias | Discussion, Final paragraphs |
| Interpretation | 20 | Give a cautious overall interpretation of results considering objectives, limitations, multiplicity of analyses, results from similar studies, and other relevant evidence | Discussion, Final paragraphs |
| Generalisability | 21 | Discuss the generalisability (external validity) of the study results | Discussion, Final paragraphs |
| Other information | | |  |
| Funding | 22 | Give the source of funding and the role of the funders for the present study and, if applicable, for the original study on which the present article is based | End of manuscript (Funding statement) |

*Give information separately for exposed and unexposed groups.

**Note:** An Explanation and Elaboration article discusses each checklist item and gives methodological background and published examples of transparent reporting. The STROBE checklist is best used in conjunction with this article (freely available on the Web sites of PLoS Medicine at http://www.plosmedicine.org/, Annals of Internal Medicine at http://www.annals.org/, and Epidemiology at http://www.epidem.com/). Information on the STROBE Initiative is available at www.strobe-statement.org.
